# Effects of Onion Peel Inclusion on In Vitro Fermentation, Methane and Carbon Dioxide Emissions, and Nutrient Degradability in Dairy Cow Diets

**DOI:** 10.3390/ani15070969

**Published:** 2025-03-27

**Authors:** Lydia K. Olagunju, Oludotun O. Adelusi, Peter A. Dele, Yasmine Shaw, Rosetta M. Brice, Oluteru E. Orimaye, Jorge A. Villarreal-González, Hye Won Kang, Ahmed E. Kholif, Uchenna Y. Anele

**Affiliations:** 1 Department of Animal Sciences, North Carolina Agricultural and Technical State University, 1601 East Market Street, Greensboro, NC 27411, USA; lkolagunju@aggies.ncat.edu (L.K.O.); ooadelusi@aggies.ncat.edu (O.O.A.); delepa@funaab.edu.ng (P.A.D.); yashaw@aggies.ncat.edu (Y.S.); rosettab101@gmail.com (R.M.B.); oeorimaye@aggies.ncat.edu (O.E.O.); villagon7@gmail.com (J.A.V.-G.); aekholif@ncat.edu (A.E.K.); 2 Department of Family and Consumer Sciences, North Carolina Agricultural and Technical State University, 1601 East Market Street, Greensboro, NC 27411, USA; hkang@ncat.edu

**Keywords:** feed additives, fermentation, gas production, nutrient disappearance, onion peel

## Abstract

Enteric greenhouse gas (GHG) emissions represent a major challenge in livestock production, contributing significantly to global methane output. Various strategies have been explored to mitigate these emissions, including dietary modifications, feed additives, and genetic improvements. In the present study, the focus was on onion peel (OP), a byproduct of the onion processing industry that has shown promise as a natural feed supplement with potential methane-reducing properties. We evaluated the effect of different inclusion levels of OP at 2.5% (OP2.5), 5% (OP5), 7.5% (OP7.5), and 10% (OP10) on the in vitro fermentation of two diets: a total mixed ration referred to as high concentrate (HC), and corn silage referred to as high forage (HF). A 48 h batch culture experiment using a 2 × 3 × 5 factorial arrangement was conducted to assess total gas production (GP), methane (CH_4_), carbon dioxide (CO_2_), ammonia (NH_3_), and hydrogen sulfide (H_2_S) concentrations, and nutrient degradability. Measurements were taken at 6 h, 24 h, and 48 h of incubation. Significant additive × diet interactions were observed for most of the parameters. The HC diet produced more gas but less CH_4_, CO_2_, NH_3_, and H_2_S compared to the HF diet (*p* < 0.05). At 24 h of incubation, the OP at all levels increased CH_4_, CO_2_, NH_3_, and H_2_S concentrations in the HF diet (*p* < 0.05). The OP2.5 treatment had the lowest (quadratic effect, *p* < 0.05) degradable dry matter (*d*DM) in the HC diet, while the OP linearly (*p* < 0.001) increased degradable acid detergent fiber (*d*ADF) in both diets. The lowest total volatile fatty acids (VFA) and acetate (quadratic effect, *p* = 0.027) were observed with the OP5 treatment in the HC diet, while OP5, OP7.5, and OP10 had lower total VFA concentration in the HC diet. At 48 h of incubation, the OP7.5 treatment increased (*p* < 0.05) GP and CH_4_ and CO_2_ production in the HC diet. However, the OP5 treatment had the lowest CH_4_ production (quadratic effect, *p* = 0.027) in the HF diet. The highest *d*DM was observed with OP7.5 treatment (quadratic effect, *p* = 0.038) in the HC diet with lower values noted at different inclusion levels in the HF diet. Inclusion of OP had no effect on total VFA, and individual VFA in both diets. In conclusion, OP supplementation is more suitable for HF diets than HC diets. A 5% inclusion level is recommended to decrease ruminal CH_4_production and improve nutrient degradability.

## Simple Summary

Exploring organic or natural feed additives has recently gained more attention. One category of these recommended feed additives is phytogenic feed additives, which are rich in secondary metabolites. This current study evaluated the effects of different levels of onion peel (OP) on two different diets for dairy cows: a high-concentrate diet and a high-forage diet. Results showed an improvement in fermentation parameters and modulation of fiber degradability in both diets. Incorporating 5% of OP in the forage-based diet is recommended as the optimal inclusion level to decrease ruminal methane production and improve nutrient degradability.

## 1. Introduction

Livestock production is considered one of the main producers of greenhouse gases (GHG) because of ruminal fermentation. Livestock production accounts for about 75% of agricultural GHG emissions, resulting in approximately 10–12% loss of digested gross energy [1]. Increasing livestock production will likely result in a corresponding increase in GHG emissions; however, animal production/productivity can be increased without an increase in total GHG emissions if GHG intensity is reduced sufficiently to offset the increase in total animal product output [2]. Animal nutritionists have evaluated several strategies to reduce GHG emissions with special concern on methane (CH_4_) production [3,4]. Phytogenic feed additives, which are rich in biologically active components, have been evaluated for their potential in sustainable animal production, yielding mixed results [1,5].

Onion peel (OP; *Allium cepa*), often discarded as agricultural byproducts, has garnered increasing attention as a potential feed additive [6]. Additionally, OP is a sustainable, cost-effective feed ingredient, as it is widely available and typically underutilized. It is estimated that about 20–30% of the total weight of onions consists of outer skins, which translates to a significant volume of OP that could be repurposed for livestock feed [7]. In the United States, approximately 3 million tons of onions are produced annually, leading to a substantial amount of OP waste [8]. Globally, China leads in annual onion production with 93.23 million tons, followed by India (19.42 million tons), Egypt (3.12 million tons), the USA (3.03 million tons), and Iran (2.35 million tons). Among all producers, European countries collectively generate 0.6 MT of OP waste each year, and a considerable portion of this is discarded as waste, providing a vast and largely untapped source of biomass that could be utilized as a feed additive [8].

The use of plant phytochemicals (i.e., secondary metabolites) to improve livestock productivity has generated renewed interest in recent times [1,9]. Studies have shown that phytochemicals contain a wide variety of phytogenic compounds, with little or no residual effect, and they have antimicrobial effects on ruminal methanogens [10,11]. Onion peels are rich in flavonoids (e.g., quercetin-3-glucoside), polysaccharide, polyphenol (e.g., quercetin, kaempferol, and anthocyanins), sulfur-containing compounds (e.g., thiosulfates and allicin), saponins, and essential oil. These compounds have shown various beneficial effects in animal nutrition, including strong antioxidant properties and antimicrobial activities [1]. Moreover, the bioactive compounds and functional groups such as terpenoids, phenolics, flavonoids and flavanols, and phenols have potent antimicrobial activities that could modify ruminal fermentation [12,13,14]. The presence of such phytochemicals may alter ruminal fermentation, decrease CH_4_ and other GHGs, and enhance nutrient degradability [6]. While other phytogenic additives, such as garlic, turmeric, and moringa, have been widely researched for their potential to enhance rumen fermentation and reduce CH_4_ emissions, OP has been relatively underexplored in the context of livestock feeding [6,15]. Previous studies have primarily focused on the essential oil components of onion and garlic, often overlooking the potential benefits of OP itself. Given its rich concentration of bioactive compounds, OP represents an untapped resource that could offer unique advantages over more commonly studied phytogenic additives, particularly in terms of improving fermentation efficiency and reducing GHG emissions in ruminants [15,16]. The administration of onion extract at 1, 3, 5, 7, or 9% to a timothy-based diet did not affect DM degradability and linearly decreased CH_4_ emission and acetate concentration [13].

Despite the growing interest in phytogenic feed additives, there is a clear gap in the literature regarding the specific effects of OP on in vitro ruminal fermentation and nutrient degradability. Most research has focused on other plant-based additives, but few studies have evaluated OP, especially in comparison to other phytogenic feed additives such as high-fiber or high-concentrate diets. Therefore, the objectives of this study are to fill this gap by investigating the effects of different levels of OP on in vitro gas production (GP), nutrient disappearance, GHG emissions [CH_4_, and carbon dioxide (CO_2_)], along with ammonia (NH_3_) and hydrogen sulfide (H_2_S), and total volatile fatty acids (VFAs) and their molar proportion of two diets rich in concentrates or forage using the in vitro batch culture technique. It is hypothesized that OP contains bioactive compounds that affect ruminal fermentation for better feed digestion and decrease GHG production.

## 2. Materials and Methods

### 2.1. Dietary Substrates

Two dietary substrates were collected from the North Carolina A&T State University Farm, and they were corn silage identified as high-forage (HF) and a total mixed ration (TMR) identified as high-concentrate (HC) diets (Table 1). The TMR consists of grain products, processed grain byproducts, plant protein products, roughage products, molasses products, and multi-vitamins and mineral supplements. Diets were dried in a forced air oven at 55 °C for 24 h and milled through a 2 mm sieve, and used as substrates for the in vitro batch culture.

### 2.2. Animal Care and Feeding

All animal procedures and uses were approved by the North Carolina Agricultural and Technical State University Institutional Animal Care and Use Committee (IACUC). This study was conducted at North Carolina Agricultural and Technical State University Dairy Research and Training Facility (NCAT DRTF; Greensboro, NC, USA). Three cannulated cows were observed daily for health problems and treated according to routine management practices at the DRTF maintained under IACUC-approved protocol LA21-009. Cows were fed corn silage and TMR ad libitum following NRC recommendations [17]. Water was provided to the cows at all times. The feeds were the same as those evaluated in the present experiment.

### 2.3. Treatments and In Vitro Batch Culture

Prior to the start of the experiment, 100 mL sample bottles were washed thoroughly and allowed to dry before use. Ankom F57 fiber filter bags (Ankom Technology Corp., Macedon, NY, USA) were labeled and soaked in acetone in a fume hood for about 30 min. Thereafter, they were transferred into the oven at 55 °C and dried to constant weight. Upon retrieving the bags from the oven, they were placed in a desiccator for about 10 min and then weighed with the same analytical scale and recorded accordingly.

The treatments were diets without OP administration (Control) or administered with OP at 2.5% (OP2.5), 5% (OP5), 7.5% (OP7.5), or 10% (OP10). A total of four replicates were prepared for each treatment. The OP levels were applied to both diets. The yellow variety of onion (*Allium cepa* L.) was used for this experiment and the peels were cleaned, air dried under shade at 30 ± 2 °C for 5 days, and then milled through a 2 mm sieve of a Retsch miller (model SM 100; Retsch GmbH, Haan, Germany). Approximately 0.5 ± 0.05 g of the treatments were individually weighed with an analytical scale (model VWR-224AC; VWR International, Radnor, PA, USA) directly into Ankom bags (57; Ankom Technology Corp, Macedon, NY, USA) and sealed using a heat impulse sealer (Model # AIE—200HR, American International Electric, City of Industry, CA, USA) before being inserted into 100 mL serum bottles (Cat# 223747; Wheaton Science Products, Milville, NJ, USA). There were four replicates (bottles) prepared for each treatment and incubated for 6, 24, and 48 h.

A detailed description of the batch culture study was described by Anele et al. [18]. Ruminal contents were collected from three Holstein Friesian cannulated cows fed the same diet (corn silage and TMR) used as substrates in the current study to minimize the potential variations in rumen inoculum. Prior to the collection of ruminal contents, hot water was used to pre-warm the container. Collected ruminal contents were squeezed through 4 layers of cheesecloth, placed inside an insulated thermos, and covered immediately after collection to maintain the normal rumen temperature and transported immediately to the Animal Nutrition Laboratory at NC A&T State University. Containers were placed in an anaerobic chamber containing CO_2_. Artificial saliva was prepared according to McDougall’s buffer recipe containing (per L) 9.83 g NaHCO_3_, 3.69 g Na_2_HPO_4_, 0.60 g KCl, 0.47 g NaCl, 0.30 g (NH_4_)_2_SO_4_, 0.061 g MgC_l2_.6H_2_O, and 0.0293 g CaC_l2_.2H_2_O. The buffer was maintained in a water bath at 39 °C. The buffer and ruminal fluid were mixed at 3:1 (*v*/*v*) and the pH was measured using a benchtop pH meter (model B10P, VWR International, Radnor, PA, USA). Afterward, 60 mL of the artificial saliva and ruminal liquor were dispensed into the serum bottles containing the substrates [18]. The serum bottles were capped with butyl rubbers and crimped with aluminum seals and placed inside an incubator equipped with an orbital shaker and set to 39 °C and 125 rpm for 48 h. Using a pressure transducer, the accumulated headspace gas pressure was measured at each of the three time points. Blanks were included to correct for the production of gas from the buffered inoculum. Corrected gas pressure values were used to estimate the GP [19,20]. All treatments were evaluated in two separate runs, with 4 replicates in each run. In each incubation run, 4 bottles with only the buffered inoculum (blanks) were also included to establish the baseline fermentation GP.

After the gas pressure and analysis readings, the liquid content of each bottle was transferred into centrifuge tubes and centrifuged for 15 min at 10,000 rpm. The filter bags in the sample bottles were removed and thoroughly rinsed under cold water until the water ran clear. The bags were oven-dried for 48 h at 55 °C. The degradation of DM, neutral detergent fiber (NDF), acid detergent fiber (ADF), and acid detergent lignin (ADL) was calculated by subtracting the dried residue weight from the initial weight of dried substrate after each analysis, as follows [21]: $\mathrm{Nutrient}\;\mathrm{degradation}=\frac{\mathrm{Initial}\;\mathrm{nutrient}\;\mathrm{weight}-\mathrm{Final}\;\mathrm{nutrient}\;\mathrm{weight}}{\mathrm{Initial}\;\mathrm{nutrient}\;\mathrm{weight}}\times 100$, and expressed as *d*DM, *d*NDF, *d*ADF, and *d*ADL, respectively.

### 2.4. Individual Gases

The GHG (CO_2_ and CH_4_) in addition to NH_3_ and H_2_S concentrations were estimated using a portable gas analyzer (Biogas 5000, Landtec, Dexter, MI, USA). The gas analyzer was calibrated per manufacturer’s instruction. An aliquot of gas from the samples was introduced into the analyzer with the aid of a 22 mm gauge needle attached to the end of the inlet Tygon tube. Between each sampling, the unit was purged to eliminate any residual gas from the previous sampling. Blanks were included to correct for the concentration of the individual gases.

### 2.5. Volatile Fatty Acid

The preserved rumen fluid samples were thawed and centrifuged at 10,000× *g* for 15 min at 4 °C, and the VFA concentration was analyzed as described by Olagunju et al. [22]. Gas chromatography described by Olagunju et al. [22] with flame ionization detection (FID) was used to quantify the VFA concentration. An internal standard mixture of metaphosphoric acid and crotonic acid (trans-2-butenoic acid) was employed, while acetic (C_2_), propionic (C_3_), butyric (C_4_), isobutyric (iso-C_4_), valeric (C_5_), and isovaleric (iso-C_5_) acid served as quantitative external standards [23]. The sample injection volume was set at 1 μL while maintaining a split ratio of 1:12. The injector port was kept at a constant temperature of 250 °C. Helium was used as the carrier gas at a flow rate of 1 mL/min, facilitating the efficient transport of the sample through the GC column. A temperature gradient was employed in the oven to optimize the separation of the analytes. Initially, the oven temperature was set at 120 °C for 0.8 min, followed by a controlled increase of 8 degrees per minute until 140 °C was reached. The oven was maintained at 140 °C for 1.8 min. The detector temperature was maintained at 280 °C. The FID operation was supported by a controlled flow of hydrogen and air gases with flow rates of 30 mL/min and 400 mL/min, respectively. Additionally, N was used as a make-up gas at a flow rate of 25 mL/min, ensuring a stable baseline and consistent detector performance. Blanks were included to correct for the total and individual VFA.

### 2.6. Chemical Analysis

The dietary samples were analyzed for DM (#930.15), N (#954.01), and ether extract (EE; #920.39) according to AOAC [24]. Nitrogen was determined using an organic elemental analyzer (2400 CHNS, PerkinElmer, Waltham, MA, USA). Ether extract was determined using the Ankom XT15 (Ankom, Macedon, NY, USA) extractor. Neutral detergent fiber and ADF were analyzed using an Ankom 200 Fiber Analyzer (Ankom, Macedon, NY, USA). The NDF content was determined as described by Van Soest et al. [25] using a heat-stable amylase with sodium sulfite. Acid detergent fiber was determined according to AOAC [24] (method 973.18). Acid detergent lignin was determined by soaking in concentrated sulfuric acid based on Ankom Technologies analytical methods. The concentrations of non-fiber carbohydrates (NFC = 100 − NDF − CP − EE − ash), cellulose (ADF−ADL), hemicellulose (NDF−ADF), and organic matter (OM = 1000 − ash) in feed ingredients and feces were calculated.

### 2.7. Statistical Analysis

Before analysis, the Shapiro–Wilk test was used to assess the normality of all dependent variables, and none showed significant deviations from normality. The data generated were analyzed using the GLM procedure of SAS 9.4 (SAS Inst., Inc., Cary, NC, USA) in a 2 × 3 × 5 factorial arrangement. The OP levels were applied to one HC and also to an HF diet. Treatment effects and interactions were examined using the probability of difference (PDIFF) option of the least squares means statement in the MIXED procedure of SAS. Polynomial (linear and quadratic) contrasts (adjusted for the equal spacing of treatments) were used to examine dose responses to increasing doses of OP. The means were separated using Tukey’s multiple comparisons test.

## 3. Results

### 3.1. Total and Individual Gas Concentrations

Time (*p* < 0.001), diet (*p* < 0.05), and time × diet (*p* < 0.05) interaction affected GP, CH_4_, CO_2_, NH_3_, and H_2_S, while additive × time × diet interactions were observed for GP, CH_4_, and CO_2_ (Table 2). The HC diet with different treatments and at each measurement time produced more gas and less CH_4_, CO_2_, NH_3_, and H_2_S gases compared to the HF diet (*p* < 0.05), with gradual increases in total GP, CH_4_, CO_2_, NH_3_, and H_2_S concentrations with increasing incubation hours (*p* < 0.001). At 6 h of incubation, additives did not affect GP, CO_2_, and H_2_S production in the two diets; however, the administration of the OP7.5 treatment to the HC diet had the highest (quadratic effect, *p* = 0.026) CH_4_ compared to the control. In contrast, the OP2.5 treatment had the lowest (quadratic effect, *p* = 0.026) CH_4_ production in the HF diet (*p* = 0.026). Moreover, the lowest (*p* = 0.019) NH_3_ was observed with the inclusion of OP10 in the HC diet. Both OP7.5 and OP10 additives had the highest NH_3_ production, while the lowest NH_3_ in the HF diet (quadratic effect, *p* = 0.019) was observed with the OP2.5 treatment. At 24 h of incubation, the administration of the OP2.5 additive in the HC diet had the highest (quadratic effect, *p* < 0.05) GP, CH_4_, CO_2_, NH_3_, and H_2_S concentrations, while the OP10 treatment decreased them. In the HF diet, all levels of the additives increased (*p* < 0.05) GP, CH_4_, CO_2_, NH_3_, and H_2_S production. At 48 h of incubation, the administration of the OP7.5 additive to the HC diet resulted in the highest (*p* < 0.05) GP, CH_4_, CO_2_, NH_3_, and H_2_S values. However, the OP5 treatment had the lowest CH_4_ and CO_2_ emissions in the HF diet (quadratic effect, *p* < 0.05).

### 3.2. Nutrient Disappearance

Nutrient degradability was affected by time (*p* < 0.001), while fiber degradability was affected by diet (*p* < 0.001) (Table 3). A significant additive × time × diet interaction was observed with *d*ADF. At 6 h of incubation, the inclusion of OP10 to the HC diet resulted in the lowest *d*DM (quadratic effect, *p* = 0.039), while the administration of OP2.5 additive to the HF diet led to the highest *d*DM. Linear increases (*p* = 0.001) in *d*ADF were observed with the administration of OP in both diets. However, all inclusion levels of OP increased (quadratic effect, *p* = 0.018) *d*ADL in the HC diet, with the lowest *d*ADL noted in the HF diet with the OP7.5 treatment. At 24 h of incubation, the administration of the OP2.5 additive in the HC diet had the lowest *d*DM (quadratic effect, *p* < 0.01), while the additives linearly (*p* < 0.001) increased *d*ADF in both diets. The lowest *d*ADL was observed with the inclusion of the OP10 treatment in the HF diet (quadratic effect, *p* = 0.47). At 48 h of incubation, the highest *d*DM was observed with the OP7.5 additive (quadratic effect, *p* = 0.038) in the HC diet, while lower values were observed in the HF diet regardless of inclusion level. A linear decrease (*p* = 0.015) in *d*NDF was observed in both diets, along with a quadratic increase (*p* = 0.023) in *d*ADF with the OP10 treatment in the HF diet. Moreover, additives linearly increased (*p* = 0.025) *d*ADL in both diets.

### 3.3. Volatile Fatty Acids Production

Only an additive × time × diet interaction was observed with C_4_ concentration (Table 4). However, time affected the concentrations and proportions of total and individual VFA (*p* < 0.001), while diets affected the concentrations of total, C_3_, C_4_, C_5_, and iso-C_5_, and proportions of C_2_, C_3_, C_4_, C_5_, and iso-acids. At 6 h of incubation, additives did not affect the concentrations of C_5_, iso-C_4_, and iso-C_5_; however, the additives linearly affected (*p* < 0.05) the proportions of all measured individual VFAs. However, additives linearly decreased the concentrations of total VFA (*p* = 0.029) and C_2_ (*p* = 0.028) with an increase in C_2_ concentration with the administration of OP2.5 in the HF diet. The highest C_3_ production was observed (quadratic effect, *p* = 0.015) with the control of the HF diet, while all inclusion levels of the additive reduced C_3_ in the HC diet, and OP5 reduced C_3_ in the HF diet.

At 24 h of incubation, additives did not affect the concentrations of iso-C_4_ and iso-C_5_, or the proportions of all individual VFAs. The OP5 additive had the lowest total VFA and C_2_ (quadratic effect, *p* = 0.027) in the HC diet, while the OP5, OP7.5, and OP10 additives had the highest total VFA in the HF. The OP treatment at all inclusion levels increased C_2_ (*p* = 0.032) and C_3_ (*p* = 0.039) in the HF diet. The highest C_4_ concentration was observed (quadratic effect, *p* = 0.014) with the administration of OP2.5 to the HF diet, while the OP10 treatment resulted in the lowest C_4_ concentration in the HC diet.

At 48 h of incubation, the OP treatment at all inclusion levels in both diets did not affect the concentrations or proportions of total VFA, C_2_, C_3_, iso-C_4_, and iso-C_5_; however, C_5_ concentration decreased in both diets.

## 4. Discussion

### 4.1. Total and Individual Gas Concentrations

Significant additive × diet × incubation time interactions were observed for most measured parameters indicating that it is essential to know the optimal level of OP administration with each diet (i.e., HC and HF diets). Expectedly, the HC diet produced more gas compared to the HF diet, indicating a higher fermentability of the HC diet due to lower lag time of GP with the HC diet [6]. Generally, HC diets contain more fermentable materials compared to the HF diets [26]; however, in the present study, both diets contained almost the same NFC and hemicellulose concentrations. The higher fermentability of the HC diet compared to the HF diet, despite similar NFC and hemicellulose concentrations, can be attributed to differences in fiber composition, protein content, and carbohydrate availability. The HC diet has lower NDF (32.0% vs. 44.7%), ADF (13.7% vs. 21.3%), and cellulose (10.8% vs. 19.6%), making it more readily fermentable, whereas the HF diet contains a greater proportion of structural carbohydrates that require extensive microbial breakdown [6]. Although the HC diet has higher ADL (2.86% vs. 1.67%), its overall fiber content is much lower, reducing the inhibitory effect of lignin on digestion. Additionally, the CP content is significantly higher in the HC diet (16.6% vs. 6.72%), which supports microbial growth and enhances fermentation efficiency.

While NFC levels are similar (39.2% vs. 38.4%), the HC diet likely contains more rapidly fermentable starches, providing a more accessible energy source for rumen microbes, whereas the HF diet has a larger proportion of structural carbohydrates within plant cell walls, slowing fermentation [27]. The higher ash content in the HC diet (6.6% vs. 4.2%) also suggests potential differences in mineral composition that could influence microbial activity [28]. Overall, the HC diet’s lower fiber content, higher protein availability, and greater accessibility of fermentable carbohydrates contribute to its higher fermentability compared to the HF diet.

During the first hours of incubation (i.e., 6 h), additives did not affect GP from the two diets, indicating that the microbes needed more time to be positively or negatively affected by the OP. However, after 24 h of incubation, the administration of OP2.5 treatment to the HC diet and all levels of OP in the HF diet increased GP. The effect of OP was greater with the HF diet than the HC diet, indicating that OP may be a more suitable strategy for fibrous diets rather than concentrated diets.

Interestingly, increasing the level of OP to 10% in the HC diet decreased GP, which may be related to increasing the level of phytochemicals as OP level increased. High levels of phytochemicals negatively affect ruminal microbial activity and growth. Onion peels contain high levels of allicin and kaempferol (0.1–0.5%, DM basis), which have been previously reported to have antibacterial activity [29] against ruminal bacteria [30]. At the end of incubation at 48 h, OP7.5 increased GP in the HC diet. Onion is rich in a flavonoid compound (kaempferol) that has high antimicrobial effects on ruminal microbes (e.g., *Fibrobacter succinogenes*, *Ruminococcus flavefaciens*, and *Butyrivibrio fibrisolvens*) at high levels [31,32], as seen from the results of GP, nutrient degradability, and VFAs. The quadratic response of GP to increasing OP levels may be attributed to microbial adaptation or the inhibitory effects of higher phytochemical concentrations [33]. At lower levels, OP might enhance microbial activity, leading to increased GP. However, at higher concentrations, the phytochemicals in OP could inhibit microbial function, resulting in a decrease in GP after an initial increase. This suggests a dose-dependent response, where optimal OP levels support microbial growth, but excessive concentrations could have negative effects on fermentation.

Reducing CH_4_, CO_2_, NH_3_, and H_2_S concentrations could reduce energy loss from ruminants, contributing to improving animal productivity. In the present experiment, higher values of these gases were reported for the HF diet compared to the HC diet, which is consistent with previous studies [34]. Li et al. [34] reported that fiber-degrading bacteria are positively correlated with CH_4_ production in sheep as ruminal microflora degrade dietary fibers to produce CH_4_ [34]. During the first hours of incubation (i.e., 6 h), the OP7.5 treatment increased CH_4_ in the HC diet, while the OP2.5 additive decreased CH_4_ production in the HF diet, indicating the importance of defining the level of OP administration based on the diet. The observed effects were based on diet type, other factors in diets, and the level of phytochemicals in the OP. The observed increase in CH_4_, CO_2_, and H_2_S productions after 24 h of incubation with the inclusion of the OP2.5 treatment in the HC diet and all levels of OP in the HF diet could be due to the extended exposure of the ruminal microflora to the OP. The same trend was observed after 48 h of incubation, where OP7.5 increased CH_4_, CO_2_, NH_3_, and H_2_S concentrations in the HC diet; meanwhile, OP5 decreased CH_4_ and CO_2_ productions in the HF diet. Onion peels are rich in allicin and other metabolites, which may also influence the activity and diversity of rumen microorganisms. Consequently, the response to the OP treatment differed between the HC and HF diets, indicating that OP could be used as a sustainable strategy to reduce CH_4_ and CO_2_ productions from animals fed HF diets, which are well known to produce more CH_4_ than HC diets. This is a positive development for the use of an HF diet for ruminant feeding, which is relatively cheaper compared to a higher quality diet.

Reducing CH_4_ production with the administration of OP in the HF diet could be related to the presence of flavonoids in OP, which are CH_4_-suppressing compounds [6,32]. Flavonoids and sulfur compounds interact with rumen microbes in various ways, influencing microbial populations and fermentation processes. Flavonoids exhibit antimicrobial properties that can inhibit methanogenic archaea, potentially reducing CH_4_ production [35]. Eom et al. [13] observed linear decreases in CH_4_ emission and methanogenic archaea abundance at 12 h incubation with the administration of onion extract. Additionally, OP is rich in allicin, a main active compound in onion, with a high ability to inhibit thiol enzyme reactivation that results in inhibiting methanogenic archaea activity and CH_4_ production [16]. Additionally, onions contain organosulfur compounds which could inhibit HMG-CoA reductase that catalyzes the synthesis of isoprenoid units in the membranes of methanogenic archaea, resulting in a reduction in CH_4_ emission. The positive effects of OP on ruminal C_3_ and C_4_ cannot be ignored as a possible reason for the change in CH_4_ concentration, as C_3_ and C_4_ are used as H-absorbing agents to directly inhibit CH_4_ production [36].

Increasing the level of OP to 10% (i.e., OP10) decreased NH_3_ produced in the HC and HF diets at 6 h of incubation, while at 24 h of incubation, the OP increased NH_3_ productions, indicating an increase in CP degradation, and consequently, more N will be available for ruminal microbes to synthesize microbial protein [37]. Yaxing et al. [37] observed that onion essential oil increased the concentration of proteinase in the rumen. Similar results were also reported by others [31,38]. In previous studies using allicin-containing plant extracts, Busquet et al. [16] reported an increase in ruminal NH_3_ concentration.

Recently, Olagunju et al. [6] evaluated the combined effects of mannan oligosaccharide and OP on ruminal in vitro fermentation using the same diets (HC and HF) as in the present study. At 6 h of incubation, the additives had no significant impact on GP or NH_3_ and H_2_S concentrations. However, the highest CH_4_ production was observed when 2% of the HC diet was supplemented with mannan oligosaccharide and OP at a 1:3 ratio. The same treatment resulted in the lowest CH_4_ production in the HF diet. After 24 h of incubation, treatments did not affect CO_2_, NH_3_, or H_2_S concentrations. The highest total GP was recorded with the mannan oligosaccharide and OP mixture at a 1:2 ratio, while the lowest was observed in the OP-supplemented HF diet. Additionally, the inclusion of mannan oligosaccharide and OP in the HC diet reduced CH_4_ production, while OP supplementation in the HF diet resulted in the lowest CH_4_ production across all the treatments.

### 4.2. Nutrient Disappearance

At 6 h of incubation, increasing the level of OP to 10% decreased the *d*DM in the HC diet, while a lower level of OP (OP2.5 treatment) was enough to decrease *d*DM after 24 h, and at the end of incubation (after 48 h), the OP7.5 increased *d*DM in the HC diet. A different pattern was observed with the HF diet, where OP positively affected nutrient digestibility. This supports the potential use of OP as a feed additive in HF diets, though further research is needed to strengthen this recommendation. The OP2.5 treatment had the highest *d*DM, and all levels of OP improved *d*ADF in both diets and at all incubation times. However, the highest level of OP (i.e., OP10 treatment) decreased *d*NDF in both diets and increased *d*ADF in the HF diet at the end of incubation due to possible negative effects of phytochemicals in the OP on ruminal microbes [33]. Higher levels of phytochemicals have antimicrobial effects of allicin and kaempferol on ruminal microbes [29,39]. At suitable levels of OP, phytochemicals may enhance the activity of ruminal microbes responsible for fiber degradation [13,40]. Sulfur compounds at high levels can lead to the accumulation of toxic levels of H_2_S, which may inhibit certain microbes and reduce fiber degradation, ultimately decreasing feed digestibility and energy production [41]. However, moderate sulfur levels are beneficial for fiber digestion and microbial growth [41]. Eom et al. [13] noted increased abundance of cellulolytic bacteria (*Ruminococcus albus*, *Fibrobacter succinogenes*, and *Ruminococcus flavefaciens*) with addition of 7% onion extract at 24 h incubation. Moreover, Yaxing et al. [37] stated that onion essential oil increased ruminal cellulase, α-amylase, and proteinase activity and the concentrations of *Prevotella* and *Prevotellaceae*_UCG-003, which contribute to the production of cellulase, α-amylase, and proteinase. Additionally, Ma et al. [31] reported that the administration of allicin (an active component in onion) to the diet of ewes increased fiber digestibility by increasing the number of cellulolytic bacteria (*Fibrobacter succinogenes*, *Ruminococcus flavefaciens*, and *Butyrivibrio fibrisolvens*) in the rumen. Consistent with the present findings, Olagunju et al. [6] examined the combined effects of mannan oligosaccharide and OP on ruminal in vitro fermentation of HC and HF diets. After 6 h of incubation, *d*NDF was higher in the HF diet compared to the HC diet, with no other observed effects of mannan oligosaccharide and OP supplementation. However, incorporating mannan oligosaccharide and OP at a 1:2 or 1:3 ratio at 2% of the diet increased *d*ADF in the HC diet, while in the HF diet, OP supplementation reduced *d*ADF. Additionally, OP decreased *d*ADL in the HC diet. After 24 h of incubation, OP treatment resulted in the lowest *d*DM, though it did not affect *d*NDF within each diet.

### 4.3. Volatile Fatty Acids Production

After 6 h of incubation, OP inclusion decreased the concentration of total VFA, C_2_, and C_3_ in both diets, which confirmed our assumption that during the first hours of incubation OP had negative effects on the ruminal microflora activity, but the microbes were able to adapt to the additive with increasing incubation period. However, the negative effects of OP in the HC diet were pronounced at 24 h of incubation. Conversely, OP5, OP7.5, and OP10 treatment increased total VFA in the HF diet at 24 h of incubation. Such effects on HF diets are desirable since VFA supports about 70 to 80% of energy needs of animals [42]. All levels of OP increased C_2_ and C_3_ concentrations in the HF diet, which are very important as C_3_ supports higher milk production [43], while C_2_ supports the synthesis of milk fat [44]. Propionic acid is a gluconeogenic VFA that influences lactose biosynthesis [45] and serves as a precursor for gluconeogenesis and lactose synthesis. Additionally, milk fat can be synthesized from C_2_, resulting in increased concentrations of milk fat precursors in the blood [46].

The alterations in the total and individual VFA could be due to the stimulating action of OP on C_3_- and C_2_-producing bacteria [37], due to increasing population of amylolytic and cellulolytic bacteria in the rumen [47]. Flavonoids and sulfur compounds in OP have antioxidant effects, helping to protect beneficial microbes from oxidative stress and modulating VFA production by promoting the synthesis of propionate over acetate [35,41]. This protective effect is important because it promotes the overall health and function of the rumen microbiome, which plays a key role in fermentation and nutrient utilization in ruminants [35,41].

At the end of incubation (after 48 h), OP did not affect total and individual VFA in both diets, indicating a possible microbial adaptation or inhibitory effects of higher phytochemical concentrations. The weak effect of additives on total and individual VFA and the decrease in CH_4_ production indicates that the OP would reduce CH_4_ emission without affecting feed efficiency in ruminants. Olagunju et al. [6] investigated the combined effects of mannan oligosaccharide and OP on ruminal in vitro fermentation of HC and HF diets. After 6 h of incubation, neither diet influenced total or individual VFA concentrations. At 24 h of incubation, the additives had no effect on C_3_, the C_2_:C_3_ ratio, iso-C_4_, or iso-C_5_ concentrations. However, the highest total VFA and C_2_ production (*p* = 0.002) were observed when both additives were administered at a 1:1 ratio to the HC diet, while the lowest values were recorded for the same additive level in the HF diet. Additionally, OP supplementation in the HF diet resulted in the lowest C_4_ and C_5_ concentrations.

## 5. Conclusions

The results of the present experiment suggest that the administration of OP may be a promising strategy for improving ruminal fermentation efficiency, and reducing CH_4_ production in HF diets compared to HC diets. The recommended levels of OP are not very clear; however, OP at 5 to 10% to the HF diet (i.e., corn silage) enhanced nutrient degradability and reduced CH_4_ production, and increased total VFA, acetate, and propionate. Therefore, additional in vitro and in vivo studies are needed to clarify the underlying mechanisms of action, evaluate the long-term effects of OP on animal performance and environmental sustainability, and address potential challenges in translating these findings into practical feeding strategies.

## Figures and Tables

**Table 1:** Chemical composition (%, DM basis) of the basal substrate ^1^.

	High Forage	High Concentrate
Dry matter	96.2	95.8
Total ash	4.20	6.60
Organic matter	95.8	93.4
Crude protein	6.72	16.6
Ether extract	5.95	5.60
Non-fiber carbohydrates	38.4	39.2
Neutral detergent fiber	44.7	32.0
Acid detergent fiber	21.3	13.7
Acid detergent lignin	1.67	2.86
Cellulose	19.6	10.8
Hemicellulose	23.4	18.3

^1^ Both the HF and HC diets were incubated individually, each representing 100% of the incubated substrates.

**Table 2:** Effects of onion peel (OP) at different levels on gas (mL/g DM), CH_4_ (mg/g DM), CO_2_ (mg/g DM), NH_3_ (mmol/g DM), and H_2_S (mmol/g DM) production of high-forage (HF) and high-concentrate (HC) diets at 6, 24, and 48 h of incubation.

Diet	Incubation Time (h)	Additive ^1^	Gas	CH_4_	CO_2_	NH_3_	H_2_S
HC	6	Control	21.9	0.31	6.9	39.3	182
		OP2.5	21.4	0.31	6.5	39.2	194
		OP5	21.3	0.29	6.2	38.4	186
		OP7.5	22.6	0.36	7.0	40.4	192
		OP10	21.3	0.29	6.4	29.3	142
HF	6	Control	13.0	0.15	3.6	14.8	78
		OP2.5	14.1	0.12	3.2	12.6	61
		OP5	14.2	0.14	3.5	17.5	88
		OP7.5	12.3	0.15	3.9	20.2	103
		OP10	12.5	0.15	3.8	21.6	101
	SEM		0.73	0.017	0.32	2.69	16.1
	Linear		0.608	0.460	0.802	0.145	0.156
	Quadratic		0.876	0.026	0.068	0.019	0.347
HC	24	Control	140	5.6	45.1	178	1401
		OP2.5	159	6.4	52.8	206	1648
		OP5	137	5.1	43.6	171	1327
		OP7.5	131	4.6	41.9	1666	1224
		OP10	126	4.5	41.1	161	1198
HF	24	Control	111	4.3	36.4	144	1043
		OP2.5	118	4.6	39.3	171	1314
		OP5	116	4.6	38.6	158	1261
		OP7.5	118	4.7	39.6	157	1240
		OP10	118	4.2	38.7	149	1142
	SEM		7.0	0.31	2.41	11.1	88.1
	Linear		0.868	0.984	0.821	0.658	0.468
	Quadratic		0.015	0.027	0.012	0.037	0.034
HC	48	Control	287	12.7	94	289	2308
		OP2.5	281	12.8	93	287	2348
		OP5	294	13.4	97	296	2413
		OP7.5	316	15.0	106	315	2583
		OP10	294	12.5	96	284	2338
HF	48	Control	284	11.5	93	312	1827
		OP2.5	282	11.9	96	349.4	2223
		OP5	267	10.9	88	298	2010
		OP7.5	275	11.4	93	281	1963
		OP10	265	11.1	90	265	1946
	SEM		2.8	0.60	3.2	22.5	141.1
	Linear		0.254	0.056	0.148	0.724	0.139
	Quadratic		0.035	0.027	0.019	0.045	0.019
Pooled SEM			6.09	0.389	2.30	14.57	96.5
Pooled *p*-value							
	Linear		0.654	0.547	0.795	0.417	0.107
	Quadratic		0.031	0.039	0.018	0.025	0.033
	Time		<0.001	<0.001	<0.001	<0.001	<0.001
	Diet		<0.001	<0.001	<0.001	0.039	<0.001
	Additive × Time		0.104	0.051	0.118	0.644	0.355
	Additive × Diet		0.829	0.704	0.897	0.805	0.697
	Time × Diet		0.028	<0.001	0.026	0.026	0.001
	Additive × Time × Diet		0.004	0.010	0.008	0.100	0.193

*p*-value is the observed significance level of the *F*-test, SEM = standard error of the mean. ^1^ Diets were incubated without additives (Control treatment) or with the addition of OP at 2.5% (OP2.5 treatment), 5% (OP5 treatment), 7.5% (OP7.5 treatment), or 10% (OP10 treatment).

**Table 3:** Effects of onion peel (OP) at different levels on dry matter and fiber degradability (%) of high-forage (HF) and high-concentrate (HC) diets at 6, 24, and 48 h of incubation.

Diet	Incubation Time (h)	Additive ^1^	*d*DM	*d*NDF	*d*ADF	*d*ADL
HC	6	Control	19.3	42.3	44.0	6.61
		OP2.5	19.6	41.8	45.8	8.10
		OP5	18.3	41.2	48.0	8.05
		OP7.5	18.0	41.1	49.1	7.62
		OP10	15.8	40.6	48.9	6.96
HF	6	Control	19.4	55.2	55.3	6.70
		OP2.5	20.6	55.4	53.8	6.40
		OP5	18.8	55.5	57.0	5.58
		OP7.5	19.2	56.6	57.3	4.74
		OP10	19.7	57.8	58.8	5.27
	SEM		0.79	1.10	0.65	0.494
	Linear		0.662	0.819	0.001	0.821
	Quadratic		0.039	0.880	0.994	0.018
HC	24	Control	29.5	48.1	46.1	9.99
		OP2.5	23.7	48.6	47.5	9.94
		OP5	29.4	48.1	48.9	9.77
		OP7.5	29.1	49.2	51.0	9.47
		OP10	28.5	48.8	52.0	8.48
HF	24	Control	26.0	63.5	57.2	8.82
		OP2.5	27.0	63.0	57.9	8.53
		OP5	27.7	63.2	58.8	7.05
		OP7.5	27.4	63.2	59.6	7.48
		OP10	26.3	62.8	59.9	6.47
	SEM		1.86	0.77	0.46	0.727
	Linear		0.613	0.702	<0.001	0.569
	Quadratic		0.028	0.641	0.307	0.047
HC	48	Control	34.3	50.0	47.9	13.0
		OP2.5	34.0	49.2	49.8	13.1
		OP5	34.7	48.6	49.2	13.6
		OP7.5	35.9	47.8	48.1	14.9
		OP10	35.2	48.0	50.0	12.5
HF	48	Control	33.5	70.5	56.2	9.00
		OP2.5	32.3	66.8	56.7	9.81
		OP5	32.2	67.8	56.5	10.12
		OP7.5	32.8	67.2	58.9	9.52
		OP10	32.5	66.6	59.4	8.22
	SEM		0.50	0.81	0.89	1.510
	Linear		0.697	0.015	0.618	0.025
	Quadratic		0.038	0.070	0.023	0.757
Pooled SEM			1.20	0.91	0.69	1.006
Pooled *p*-value						
	Linear		0.702	0.195	<0.001	0.486
	Quadratic		0.165	0.196	0.192	0.848
	Time		<0.001	<0.001	<0.001	<0.001
	Diet		0.135	<0.001	<0.001	<0.001
	Additive × Time		0.178	0.209	0.026	0.813
	Additive × Diet		0.452	0.723	0.212	0.626
	Time × Diet		0.005	<0.001	0.221	0.017
	Additive × Time × Diet		0.300	0.376	0.021	0.982

*p*-value is the observed significance level of the *F*-test, SEM = standard error of the mean. ^1^ Diets were incubated without additives (Control treatment) or with the addition of OP at 2.5% (OP2.5 treatment), 5% (OP5 treatment), 7.5% (OP7.5 treatment), or 10% (OP10 treatment). *d*DM is dry matter degradability; *d*NDF is neutral detergent fiber degradability; *d*ADF is acid detergent fiber degradability; *d*ADL is acid detergent lignin degradability.

**Table 4:** Effects of onion peel (OP) at different levels on total and individual volatile fatty acid concentrations (mmol/g DM) and proportions of high-forage (HF) and high-concentrate (HC) diets at 6, 24, and 48 h of incubation.

Diet	Incubation Time (h)	Additive ^1^	Total	C_2_	C_2_ (%)	C_3_	C_3_ (%)	C_4_	C_4_ (%)	C_5_	C_5_ (%)	Iso-C_4_	Iso-C_4_ (%)	Iso-C_5_	Iso-C_5_ (%)
HC	6	Control	56.1	41.4	73.8	9.33	16.7	4.52	8.1	0.53	0.94	0.21	0.37	0.08	0.14
		OP2.5	52.4	38.2	72.8	9.01	17.2	4.48	8.6	0.52	0.98	0.20	0.38	0.08	0.15
		OP5	49.8	35.6	71.4	8.92	17.9	4.55	9.1	0.51	1.02	0.20	0.41	0.08	0.16
		OP7.5	48.8	34.4	70.3	8.89	18.3	4.71	9.7	0.54	1.12	0.21	0.43	0.08	0.17
		OP10	50.9	36.9	72.6	8.71	17.2	4.42	8.7	0.51	1.01	0.19	0.37	0.08	0.15
HF	6	Control	50.4	36.8	72.9	8.73	17.3	4.09	8.1	0.54	1.06	0.22	0.43	0.09	0.18
		OP2.5	52.0	39.0	73.0	8.33	17.3	3.87	8.1	0.50	1.03	0.21	0.43	0.09	0.18
		OP5	44.5	31.9	74.5	8.00	16.3	3.87	7.6	0.51	0.97	0.20	0.40	0.09	0.18
		OP7.5	48.2	35.1	71.4	8.39	18.0	3.92	8.8	0.50	1.15	0.21	0.45	0.09	0.20
		OP10	47.5	34.7	72.8	8.21	17.4	3.86	8.1	0.49	1.03	0.20	0.43	0.09	0.18
	SEM		2.23	2.08	0.88	0.202	0.53	0.078	0.30	0.070	0.045	0.006	0.017	0.002	0.007
	Linear		0.029	0.028	0.017	0.094	0.048	0.616	0.003	0.293	0.046	0.655	0.033	0.876	0.030
	Quadratic		0.646	0.858	0.474	0.045	0.480	0.015	0.503	0.100	0.423	0.069	0.332	0.065	0.599
HC	24	Control	76.5	48.2	62.9	16.75	21.9	10.26	13.4	0.91	1.19	0.31	0.41	0.13	0.17
		OP2.5	76.2	46.9	65.2	17.34	20.9	10.58	12.2	0.96	1.08	0.30	0.37	0.13	0.16
		OP5	72.5	45.6	61.5	15.62	22.7	9.98	14.0	0.87	1.25	0.30	0.40	0.13	0.17
		OP7.5	74.6	47.5	62.8	16.11	21.6	9.69	13.8	0.83	1.22	0.29	0.41	0.12	0.18
		OP10	76.0	49.6	63.7	15.89	21.6	9.31	13.0	0.82	1.12	0.28	0.39	0.12	0.16
HF	24	Control	67.5	43.9	65.0	12.98	19.2	9.46	14.0	0.74	1.10	0.30	0.44	0.14	0.20
		OP2.5	77.8	51.3	65.8	14.94	19.2	10.36	13.4	0.76	0.98	0.32	0.41	0.14	0.18
		OP5	72.8	47.8	65.7	14.13	19.4	9.67	13.3	0.75	1.03	0.30	0.41	0.14	0.19
		OP7.5	73.8	48.3	65.4	14.75	20.0	9.57	13.0	0.78	1.05	0.31	0.42	0.14	0.19
		OP10	72.9	48.0	65.7	14.42	19.8	9.34	12.8	0.75	1.03	0.30	0.41	0.14	0.19
	SEM		2.98	2.20	0.65	0.667	0.37	0.282	0.35	0.022	0.040	0.009	0.018	0.003	0.008
	Linear		0.712	0.744	0.944	0.571	0.469	0.951	0.599	0.581	0.504	0.923	0.767	0.421	0.413
	Quadratic		0.027	0.032	0.928	0.039	0.994	0.014	0.969	0.038	0.660	0.742	0.246	0.577	0.543
HC	48	Control	92.9	58.3	62.7	21.08	22.7	11.55	12.5	1.31	1.42	0.45	0.48	0.20	0.21
		OP2.5	89.4	56.0	63.8	20.43	22.4	11.16	11.8	1.25	1.27	0.42	0.44	0.19	0.20
		OP5	93.5	58.7	62.6	21.06	22.9	11.75	12.5	1.27	1.40	0.44	0.47	0.20	0.21
		OP7.5	95.8	60.6	62.8	21.43	22.6	11.93	12.6	1.25	1.36	0.46	0.47	0.21	0.21
		OP10	95.3	60.9	63.2	21.32	22.4	11.26	12.5	1.21	1.30	0.42	0.48	0.19	0.21
HF	48	Control	96.4	62.3	64.6	19.91	20.6	12.42	12.9	1.17	1.21	0.43	0.45	0.21	0.21
		OP2.5	93.4	59.5	63.7	19.94	21.3	12.31	13.2	1.07	1.14	0.42	0.45	0.20	0.21
		OP5	94.2	59.3	63.0	20.72	22.0	12.39	13.2	1.10	1.17	0.45	0.48	0.20	0.22
		OP7.5	90.8	59.1	65.1	19.07	21.0	11.02	12.1	1.02	1.12	0.39	0.43	0.19	0.20
		OP10	91.4	59.4	64.9	19.33	21.2	11.01	12.1	1.07	1.17	0.40	0.44	0.19	0.21
	SEM		2.17	1.83	0.80	0.58	0.54	0.293	0.28	0.034	0.043	0.015	0.014	0.005	0.005
	Linear		0.657	0.626	0.787	0.948	0.703	0.898	0.789	0.028	0.088	0.771	0.571	0.719	0.936
	Quadratic		0.312	0.256	0.337	0.952	0.289	0.761	0.454	0.210	0.787	0.248	0.583	0.269	0.946
Pooled SEM			2.49	2.04	0.78	0.522	0.49	0.239	0.310	0.024	0.043	0.011	0.016	0.003	0.007
Pooled *p*-value															
	Linear		0.252	0.164	0.071	0.937	0.065	0.964	0.117	0.019	0.915	0.886	0.155	0.451	0.366
	Quadratic		0.997	0.935	0.951	0.759	0.811	0.757	0.991	0.171	0.373	0.068	0.112	0.204	0.445
	Time		<0.001	<0.001	<0.001	<0.001	<0.001	<0.001	<0.001	<0.001	<0.001	<0.001	<0.001	<0.001	<0.001
	Diet		0.040	0.819	<0.001	<0.001	<0.001	0.027	0.295	<0.001	<0.001	0.695	0.032	<0.001	<0.001
	Additive × Time		0.187	0.170	0.060	0.240	0.338	0.011	0.002	0.019	0.081	0.297	0.522	0.629	0.215
	Additive × Diet		0.336	0.275	0.262	0.637	0.244	0.133	0.080	0.343	0.059	0.247	0.319	0.056	0.301
	Time × Diet		0.401	0.277	0.096	0.009	<0.001	0.002	0.002	<0.001	<0.001	0.015	0.001	0.004	<0.001
	Additive × Time × Diet		0.389	0.596	0.361	0.204	0.481	0.008	0.359	0.060	0.134	0.056	0.494	0.010	0.585

*p*-value is the observed significance level of the *F*-test, SEM = standard error of the mean. ^1^ Diets were incubated without additives (Control treatment) or with the addition of OP at 2.5% (OP2.5 treatment), 5% (OP5 treatment), 7.5% (OP7.5 treatment), or 10% (OP10 treatment). C_2_ is acetate; C_3_ is propionate; C_4_ is butyrate; C_5_ is valerate (mmol/g DM).

## Data Availability

Data are available within the manuscript.

## Abbreviations

The following abbreviations are used in this manuscript:

ADF	Acid detergent fiber
C_2_	Acetate
C_3_	Propionate
C_4_	Butyrate
CH_4_	Methane
CP	Crude protein
CO_2_	Carbon dioxide
*d*ADF	Degradable acid detergent fiber
*d*DM	Degradable dry matter
DM	Dry matter
*d*NDF	Degradable neutral detergent fiber
EE	Ether extract
GHG	GHG
GP	Gas production
HC	High concentrate
HF	High forage
H_2_S	Hydrogen sulfide
NDF	Neutral detergent fiber
OM	Organic matter
OP	Onion peel
NH_3_	Ammonia
VFA	Volatile fatty acids
